# Bladder cancer screening: The new selection and prediction model

**DOI:** 10.1515/med-2023-0723

**Published:** 2023-06-16

**Authors:** Vladan Radosavljevic, Natasa Milic

**Affiliations:** Military Medical Academy, Institute of Epidemiology, Crnotravska 17, 11000 Belgrade, Serbia; Institute of Medical Statistics and Informatics, Medical Faculty, University of Belgrade, 11000 Belgrade, Serbia

**Keywords:** bladder cancer, screening, model, selection, prediction

## Abstract

The objective of this study was to offer new approach for selection of persons with asymptomatic bladder cancer (BC) and highly risky persons for the BC occurrence. Also, it is a part of the BC screening protocol (study is ongoing). Study populations were 100 newly diagnosed (diagnosis maximum 1-year old) males with BC and 100 matched (by sex and age ±5 years) controls (not oncology patients from the same hospital). A hospital based, matched case–control study was done. Statistical analysis comprised of four steps: *t*-test, univariate logistic regression, multivariate logistic regression, and scoring. The fifth step comprised of two changes, deleting one variable and addition of another variable. Six variables were statistically significant: Caucasian men over 45 years age, tobacco smoking over 40 pack-years, occupational and/or environmental exposure to the proved BC carcinogens over 20 years, macrohematuria, difficulty urinating, BC in relatives up to fourth degree of kinships, and they were used for an easy and fast selection of the individuals with high risk for BC occurrence and BC asymptomatic patients (optimal selection at the population level). The final results showed highly significant probability (*p* < 0.001), with area under ROC curve of 0.913, negative predictive values of 89.7% (95% CI 10.3–100%), and a specificity of 78%. Positive predictive value was 80.5% (95% CI 19.5–100%) and a sensitivity of 91%. It is possible to recruit asymptomatic BC patients (primary prevention) by using this model, as well as persons with high risk for BC occurrence (primordial prevention). This study is the first part of the BC screening protocol and the second part of the BC screening protocol study is ongoing (urine analysis).

## 1 Introduction

Bladder cancer (BC) is among the most frequent malignancies worldwide, especially in Europe and North America, elderly men, Caucasians, smokers, and occupationally exposed people. Due to the growth of the global population, both in terms of size and age, the increase of both incidence and prevalence of BC patients may be expected [1,2,3]. Over 1.7 million people live with BC worldwide. Currently, people at the age of 60+ represent approximately 13.7% of the world population, varying from 25% in Europe to 5% in Africa. Population at the age of 60+ is rising from current over 970 million to 1.4 billion by 2030 and 2.1 billion by 2050 [4]. In less developed countries the incidence of BC is increasing because of high rate of smoking [3]. Over 95% of BCs are *transitiocellulare* pathohistological type, while the remainder up to 5% are rare subtypes such as *squamocellulare,* adenocarcinoma, sarcoma, and metastases to the bladder [2].

Worldwide, the lifetime risk of getting BC is 1.1% in men and 0.27% in women [1]. The lifetime risk is considerably higher in most developed countries, such as the US (lifetime risk in men and women is 3.9 and 1.2%, respectively) [1]. Worldwide, approximately 213,000 patients died from BC in 2020 (2.1% of all cancer sites, for men 4.4%), and incidence was over 573,000 patients (3.0% of all cancer sites, for men 3.9%). BC is more common in men than in women, with respective incidence and mortality rates of 9.5 and 3.3 per 100,000 among men, which are approximately four times higher than among women globally [5].

BC is the most expensive cancer for treatment, due to recurrent nature of disease, expensive disease monitoring, and curing by trimodal therapy: surgery, chemotherapy, and radiotherapy [3,6]. Non-muscle invasive bladder cancer (NMIBC) accounts for about 75% of newly diagnosed BCs. It is limited to mucosa (stage Ta) or submucosa (stage T1). NMIBC recurs in 50–70% of the cases. More progressive malignancy occurs in 10–15% of the cases [7].

There is no officially or widely accepted method for BC screening. Due to the low BC incidence in general population, selection of asymptomatic BC patients from the general population is the main problem for BC screening [6,8]. To the best of our knowledge, this approach was used for the first time ever. This model uses BC screening for two purposes: primordial BC prevention and primary BC prevention. Primordial prevention includes detection of persons with high risk for the BC occurrence. Primary prevention includes detection of persons with early BC (NMIBC).

Urinary biomarkers showed 12–26% false-positive results in respondents when used for BC screening, and combined with biomarkers’ limited sensitivity, could present a missed diagnosis in up to 43% respondents. The main and the most studied urinary biomarkers and their effectiveness are nuclear matrix proteins (NMP)22 Bladder Cancer ELISA-test (sensitivity of 69% and a specificity of 77%), NMP22 BladderChek tests (sensitivity of 52–59% and a specificity of 87–89%), Bladder Tumour Antigen (BTA) Stat-test (sensitivity 57–82% and a specificity of 68–93%), BTA TRAK-test (sensitivity of 66–77% and a specificity ranging from 5 to 75%), UroVysion test is a multicolour fluorescent *in situ* hybridization (FISH) assay (sensitivity ranges between 69 and 87% and a specificity between 89 and 96%), ImmunoCyt assay (uCyt+) with a sensitivity ranging from 60 to 100% and a specificity of 75–84%, URO17™ urine test (sensitivity of 100% and specificity of 96%), BLCA4 (sensitivity of 89–96% with a specificity of 95–100%), CellDetect assay (sensitivity of 84% and the specificity of also 84%), and aurora A kinase (AURKA)-FISH test (sensitivity 87% and 96.6% specificity). DNA-based urine biomarkers (DNA Methylation) are GSTP1, RARb2, APC genes (sensitivity 62% and specificity 89%), TWIST1 and NID2 genes (sensitivity 79% and specificity 63%), POU4F2 and PCDH17 genes (sensitivity 90% and specificity 94%), CFTR, SALL3/TWIST1 genes (sensitivity 84% and specificity 68%), and HDAC3 genes (sensitivity 89% and specificity 63%). Genetic alterations include both DNA mutational analysis – urinary Telomerase reverse transcriptase promoter mutations (sensitivity of 80.5% and specificity of 89.8%) and microsatellite analysis – a PCR analysis of DNA in exfoliated urine cells (sensitivity range from 72 to 97% and the specificity between 80 and 100%). Urinary tumour RNAs include MicroRNAs (miRNAs) which can be derived from a range of specimens – supernatant, urine sediment, and voided urine. The most important are 6 miRNAs = miR-187 + miR-18a + miR-25 + miR-142-3p + miR140-5p + miR204 (sensitivity 85% and specificity 87%), miR-99a + miR-125b (sensitivity 87% and specificity 81%), miR-96 + cytology (sensitivity 87% and specificity 87%), and 25 target diagnostic miRNA signature (sensitivity 87% and specificity 100%). Finally, multigene panels involve DNA, mRNA, and epigenetic targets, and the most important among them is Cxbladder with genes: IGFBP5, HOXA13, MDK, CDK1, and CXCR2 (sensitivity 82% in patients with hematuria and specificity 90%) [9].

miRNAs are single stranded, non-coding RNAs (20–25 nucleotides) that are hypothesized to regulate gene expression at the post-transcriptional level. The results of the present study indicated that miR-200, miR-145, and miR-21 may function as oncogenes and have a potential to serve as an early non-invasive diagnostic biomarkers and therapeutic targets for treatment of BC. Authors suggest that miR-200, miR-145, and miR-21 may function as diagnostic and prognostic markers as well as possible therapeutic targets for treatment of BC [10,11]. Identification of alterations in genes that are frequently mutated in BC appears to be a promising strategy for detecting disease from urine samples and reducing reliance on examination of the bladder via a telescopic camera inserted through the urethra (flexible cystoscopy has estimated sensitivity of 85% and specificity of 87%). Urinary DNA arguably yields the most robust tumour-specific information for non-invasive BC detection. Some authors described the development and validation of a non-invasive test for detection of BC based on error suppressed ultra-deep sequencing of somatic mutations in 23 genes in urinary DNA. The test has the potential to detect new cases of BC with high sensitivity and specificity [11,12].

Because of the low prevalence of BC in the general population (0.001%), even in elderly over the age of 50 (0.67–1.13%), followed by detecting a significant number of BC false positive results, mass screening is certainly not acceptable [13]. Therefore, introducing BC risk scoring in screening protocol seems to be very promising. Ideal screening candidates should be non-invasive, cheap, fast, friendly for use, highly sensitive, and highly specific.

In around 80% of BC patients the diagnosis was made at an age 65 or older, reflecting a disease course that occurs after several decades of exposure even if the exposure only lasted several years. BC is a prime candidate for prevention strategies as 80% of cases are attributable to known risk factors [2].

## 2 Patients and methods

A literature review was performed in the MEDLINE database. The focus of the literature search was for the period between January 1, 2014 and March 31, 2023, to obtain the necessary data for a retrospective analysis of the BC screening possibilities and attempts. The indicated period was reviewed because the author’s last published review article from the BC field was published in January 2014 [6].

The BC screening was the guiding question of the literature review. The types of reviewed literature were the original research articles as well as the review articles published in the English language in peer-reviewed journals and books. Criteria for inclusion in the review were the following keywords: bladder cancer, screening, cohort, research and population. The time period in which the search was carried out was between July 1, 2021 and March 31, 2023.

The Pubmed advanced search strategy was used for the article selection. Query box “All fields” was used, due to its comprehensiveness. Step one: bladder cancer AND screening = 2,139 full text references; terms to the query box: All fields. Step two: bladder cancer AND screening AND cohort = 181 full text references; terms to the query box: All fields. Step three: bladder cancer AND screening AND research = 740 full text references; terms to the query box: All fields. Step four: bladder cancer AND screening AND population = 205 full text references; terms to the query box: All fields. Two main criteria for the articles and books selection were scientific informativeness and scientific reliability. The first group consisted of a very large number of the papers that were rejected after consideration of the articles’ title. Numerous groups of articles were rejected from further review after studying the abstracts of the articles. The third group of articles was rejected after musing upon the articles’ methods and results. The fourth group of articles was discarded after detailed considering the entire article. Finally, the fifth group of articles was eliminated after reading and mutually comparing with other articles from the field.

This model has two aims: (1) selection of people with high risk for BC occurrence and (2) selection of patients with early BC. We structured interview according to both: proved BC risk factors and observing the most affected population. The focus of the interview was on Caucasian men over 45 years age, tobacco smoking, arsenic in drinking water, occupational exposure (intensity and duration), heredity (BC or other malignancy present in the first four kinship degrees), gross hematuria, genitourinary infections, benign prostate enlargement, and diabetes over 20 years duration. The remaining questions were about BC disease (time of the disease onset, clinical stadium, pathohistological findings). The total number of participants enrolled in the study was 200, and 100 of them had a history of BC. First group of 50 participants were randomly selected from January 2018 to August 2021, and the second group of 50 consecutive patients from September 2021 to December 2021. The patients were recruited from the Urology Clinic of the Military Medical Academy in Belgrade (Serbia). An experienced epidemiologist interviewed 50 patients while urologists interviewed another 50 patients. The same number of controls matched by sex and age (±5 years) were simultaneously interviewed from the same hospital as patients (rheumatology department, physical medicine and rehabilitation department, and cardiology department). At the cardiology department patients whose disease could be influenced by tobacco smoking were excluded. Statistical analysis comprised of four steps: *t*-test, univariate logistic regression, multivariate logistic regression, and scoring. The fifth step comprised of two changes, deleting one variable and addition in another variable.

The research related to human use has been complied with all the relevant national regulations, institutional policies and in accordance the tenets of the Helsinki Declaration, and has been approved by the Ethical Board of the Military Medical Academy on November 27, 2017. Before the interview, all participants provided informed consent for participation in the study.

## 3 Results

The mean age of patients in this study was 67.31 years, 83% of the patients were married.

### 3.1 Statistical analysis

Step 1. *t*-Test was applied on all variables (questions from the questionnaire) and statistically significant differences were found for level of education: high school and lower levels of education vs post-high school education or university level, *p* = 0.001; tobacco smoking over 40 pack-years vs less than 40 pack-years, *p* < 0.001; occupational exposure to the proved BC carcinogens over 20 years vs less than 20 years, *p* < 0.001; macrohematuria, *p* < 0.001; difficulty urinating, *p* < 0.001; and BC in relatives up to fourth degree of kinships, *p* = 0.002.

Step 2. Univariate logistic regressions on the previous six significant variables and results are presented in Table 1.

**Table 1:** Univariate logistic regression

Variables	*p*	OR	95% CI
Level of education	0.001	2.565	1.448–4.542
Tobacco smoking over 40 pack-years	0.000	6.303	3.374–11.775
Occupational exposure to the proved BC carcinogens over 20 years	0.000	4.758	2.131–10.626
Macrohematuria	0.000	34.810	15.836–76.517
Difficulty urinating	0.000	2.901	1.633–5.151
BC in relatives up to fourth degree of kinships	0.032	9.791	1.217–78.806

Step 3. Multivariate logistic regressions on the previous six significant variables. The results are presented in Table 2.

**Table 2:** Multivariate logistic regression

Variables	*p*	OR	95% CI
Tobacco smoking over 40 pack-years	0.000	4.696	1.966–11.218
Macrohematuria	0.000	30.101	12.544–72.232
Difficulty urinating	0.016	2.910	1.218–6.953
BC in relatives up to fourth degree of kinships	0.022	24.281	1.585–371.983

**Figure 1:**
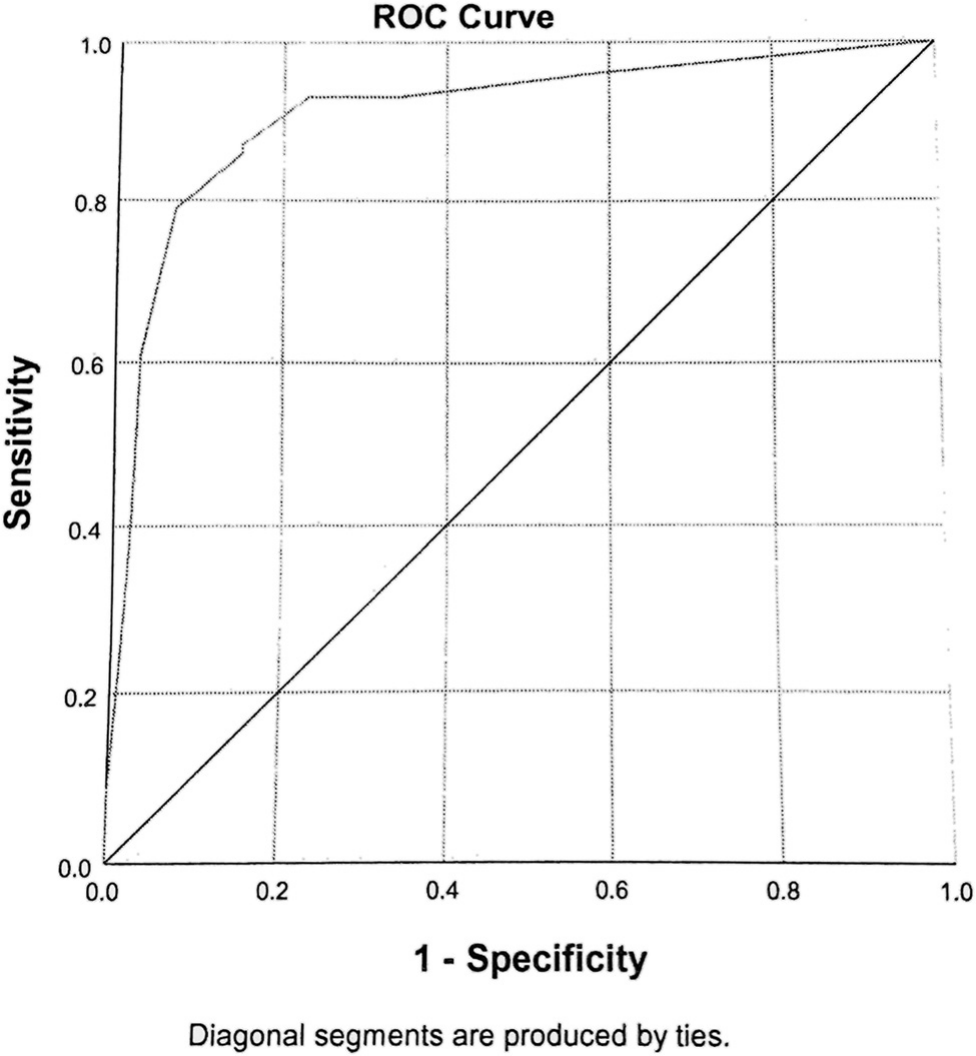
(Brief self-explanatory legend) Area under ROC curve of 0.913, NPV of 89.7% (95% CI 10.3–100%), and a specificity of 78%. PPV was 80.5% (95% CI 19.5–100%) and a sensitivity of 91%.

Step 4. Our preference was to make a method with high sensitivity. Thus, we decided to make Score 1 based on the univariate logistic regression results, e.g., based on six significant variables (Table 1). Cut-off was 10 for Score 1. Score 1 has highly significant probability (*p* < 0.001), with area under ROC curve of 0.913, negative predictive values (NPV) of 89.7% (95% CI 10.3–100%), and a specificity of 78% (Figure 1). Positive predictive values (PPV) was 80.5% (95% CI 19.5–100%) and a sensitivity of 91%.

Step 5. Very recent and comprehensive literature reconsideration strongly suggested us to make some changes in the results of the univariate logistic regression [2,11–16]. In the final model we decided to the delete variable “Level of education.” In the variable “Occupational exposure to the proved BC carcinogens over 20 years” add word environmental. Therefore, the issue should be “Occupational and/or environmental exposure to the proved BC carcinogens over 20 years” (Table 3).

**Table 3:** New selection and prediction model for BC occurrence

Variables	*P*	OR	95% CI
Tobacco smoking over 40 pack-years	0.000	6.303	3.374–11.775
Occupational and/or environmental exposure to the proved BC carcinogens over 20 years	0.000	4.758	2.131–10.626
Macrohematuria	0.000	34.810	15.836–76.517
Difficulty urinating	0.000	2.901	1.633–5.151
BC in relatives up to fourth degree of kinships	0.032	9.791	1.217–78.806

There was no statistical significance for the next variables: arsenic in drinking water ≥10 µg/L consumed over 20 years, genitourinary infections four or more times during lifetime, carcinomas of the upper urinary tract, diabetes mellitus (any type) over 20 years old diagnosis, benign tumours of the urinary bladder, and any other malignancies in relatives.

## 4 Discussion

Developing BC screening protocol is a great challenge. The time between screen detection and progression to advanced disease is a sufficiently lengthy period to allow for intervention. Some authors developed strategies for identifying high-risk populations for BC screening using the most frequent BC risk factors, and even more, defined risk score [17,8]. Screening studies have suggested a survival benefit amongst screened non-symptomatic populations with known risk factors, but it has not become a standard practice [18]. Chinese authors developed a prediction model of oesophageal cancer with good sensitivity based on eight epidemiological risk factors [19].

It is clear that a mass BC screening would only be feasible if restricted to a high-risk group [17]. Screening is the most successful method in high-risk populations and its cost efficacy may be reached if applied to a population with an incidence of BC >1–2% [8]. Risk prediction methods may optimize the universal endoscopic screening strategy, and show promising usefulness in screening practice [20].

### 4.1 The new selection and prediction model (variables)

#### 4.1.1 Caucasian men over 45 years age

BC incidence disparities between genders are well documented [21]. Reasons for sex gap in BC incidence are differences in the following variables: smoking patterns, occupational exposures, access to health care, and delayed diagnosis in women (hematuria or lower urinary tract symptoms frequently being attributed to cystitis) [22]. Nations with the highest rates of BC are identified in Europe and North America, and the disease is four times more frequent in men than in women [2]. In less developed countries there is a lack of national and/or regional population-based cancer registries [1]. Black people in North America have a lower incidence rate for BC. Some researchers reported increasing trend of BC in Asian and Central and Eastern European developing countries [21]. About 90% of BC diagnoses are made in those of age 55 and older [2]. The mean age of patients in the United States diagnosed with BC is 73 years with 90% of patients being over 55 years old [23].

We included in the study Caucasian men over age 45. The mean age of patients in this study is 67.31 years, which is in accordance with other authors.

#### 4.1.2 Tobacco smoking

The risk of BC was three to four times higher in smokers or ex-smokers than in non-smokers [2,23,24]. The population attributable risk of BC for tobacco smoking is 50–65% in men [8,23]. BC risk occurrence following tobacco smoking is second, just after lung cancer risk occurrence [2].

The older age of onset of BC suggests a latency period of approximately 30 years from the initiation of smoking to the cancer diagnosis. However, smoking cessation has been shown to reduce the risk of BC by approximately 40% within 1–4 years, and complete return to baseline risk by 20 years. A meta-analysis of 14 studies showed increased risk of BC for 22% during lifetime for secondhand smoking exposure in non-smoking respondents if compared with unexposed non-smoking population [2].

In a high-risk group with a history of smoking of ≥40 pack-years recorded 3.3% respondents with a histologically confirmed BC and another 6.6% respondents with precancerous lesions [25]. The smokers among BC patients were heavier smokers (mean 43.5 pack-years) [24,26].

Our results show statistically significant correlations in tobacco smoking over 40 pack-years, while BC occurrence is in accordance with the findings of other authors.

#### 4.1.3 Occupational exposure

According to the literature the most prominent BC carcinogens are aniline (mainly in azo dyes) and polycyclic aromatic hydrocarbons (PAHs) [6]. Chemical carcinogens also contributing to BC occurrence (needed more intensity and/or more duration of exposure) are PAHs, 2-naphthylamine, 4-aminobiphenyl, toluene, 2-chloroaniline, and metal working fluids. It should be noted that all fossil fuels and woods produce PAHs, especially during incomplete combustion. Risky occupations are tobacco workers, dye and paint workers, metal and rubber industry workers [14,22–24].

Recent articles reported that people living nearby combustion, mining, mechanical/car manufacturing establishments, and power plants had an increased BC risk, regardless of employment (environmental exposure to aromatic amines, PAHs, vehicle exhaust, and heavy metals) [27]. For example, Saginala and coauthors, reported occupational or environmental toxins accounting for an estimated 20% of all BC cases [2].

Workers exposed to aromatic amines (anilines) or PAHs have 16–23% higher risk for BC occurrence than the unexposed. Larré and coworkers reported that about 4% of BC is attributable to occupational exposure. Cumberbatch and coauthors informed that occupational carcinogen exposure accounts for approximately 5–6% of BC occurrences [8,18].

The risk of BC occurrence persists for up to 30 or 40 years after occupational exposure [8,24]. In some cases 2 years’ exposure seems to be sufficient to increase one’s BC risk decades after exposure [2].

Our results are in accordance with the previous findings.

#### 4.1.4 Heredity (BC or other malignancy present in the first four kinship degrees)

Saginala and coauthors (2022) reported that heritable genetic predispositions contributed in about 7% of BC cases. Larré and coworkers cited that heredity accounts for <2% of new BC cases, and close relatives with BC are more often in BC patients diagnosed at a younger age [8].

NAT2 is a slow acetylator which detoxifies aromatic amines, and GSTM1 is an enzyme included in the detoxification of other environmental carcinogens. Abnormalities in genes NAT2 and GSTM1 lead to longer exposure to carcinogens. Their expression through phenotype is extremely different [18,26]. But, Vickers and coworkers informed that family history did not discriminate satisfactorily, and even more family history might be dropped from the eligibility assessment [17].

This study showed statistically significant co-relations between BC occurrence in relatives up to fourth degree of kinships and BC patients.

#### 4.1.5 Hematuria

Painless hematuria is the most common but not mandatory early BC sign in ≈85% of patients [25]. Four studies, each of them included over one thousand elderly men tested for hematuria either repeatedly or subsequently reported about 0.6–1.3% BC participants. Population-based screening studies of men (age over 50 years) reported that 16–24% of respondents had hematuria, while 32% of BC patients did not have hematuria (hematuria is intermittent in many patients) [8]. In sources obtained from 2,356 screened asymptomatic men over the age of 60 years, 474 men (20%) had dipstick hematuria of which 5.3% (17/319 who agreed to further evaluation) showed asymptomatic BC [23]. Finally, macroscopic hematuria was present in 17% of cases of BC while microscopic hematuria was present in 4% of BC cases [28].

Our study showed statistically significant correlations between BC occurrence and macrohematuria.

#### 4.1.6 Limitations of the study

To the best of our knowledge limitations of this study are relatively small samples of cases (100 persons) and controls (100 persons). These numbers should be increased. Also, the presented model should be reproduced in other centres and confirmed in them, or in other studies.

## 5 Conclusion

Screening studies of high-risk populations will probably become routine and more accurate for prevention of BC, oesophageal cancer, and similar diseases with low incidence in the general population. We developed a BC prediction and selection model (described six variables) that offers physicians and public health workers an opportunity for easy and fast selection of the individuals with high risk for BC occurrence and BC asymptomatic patients (optimal selection at the population level). This study is the first part of the BC screening protocol study, and the second part of the BC screening protocol study is ongoing (simple chemical analysis of urine).
